# A Systematic Review and Meta-Analysis of Maternal Dengue Infection and Adverse Pregnancy Outcomes

**DOI:** 10.7759/cureus.96888

**Published:** 2025-11-15

**Authors:** Elaveyini U, Tharanikumar Sivakumar, Chandrasekaran Krithika, Chitathoor Sridhar, Aroshime Hercules

**Affiliations:** 1 Obstetrics and Gynaecology, Saveetha Medical College and Hospital, Saveetha Institute of Medical and Technical Sciences, Chennai, IND; 2 Oral and Maxillofacial Surgery, Meenakshi Academy of Higher Education and Research, Chennai, IND; 3 Oral Medicine and Radiology, Meenakshi Ammal Dental College, Meenakshi Academy of Higher Education and Research, Chennai, IND; 4 Internal Medicine, Meenakshi Academy of Higher Education and Research, Chennai, IND; 5 Obstetrics and Gynaecology, New Hope Hospital, Chennai, IND

**Keywords:** dengue virus (denv), miscarriage, neonatal mortality, pregnancy-related outcomes, stillbirth, systematic review and meta-analysis

## Abstract

Dengue virus (DENV) infection is endemic in many regions with high fertility rates and poses a significant public health concern during pregnancy. This systematic review and meta-analysis aimed to evaluate the association between maternal dengue infection and adverse pregnancy outcomes. A comprehensive search of PubMed, Embase, and Web of Science was conducted up to July 11, 2025. Eligible studies were comparative observational in design, with laboratory-confirmed dengue infection in pregnant women and at least one fetal or neonatal outcome reported. Outcomes of interest included stillbirth rate (SBR), miscarriage rate (MBR), preterm birth rate (PBR), low birth weight (LBW), small for gestational age (SGA), neonatal death (ND), and postpartum hemorrhage (PPH). Random-effects models were employed to calculate pooled odds ratios (OR), and the risk of bias was assessed using the Newcastle-Ottawa Scale.

A total of 35 studies encompassing approximately 65,000 pregnancies from 14 countries were included. Dengue infection during pregnancy was significantly associated with increased odds of stillbirth (OR 2.70; 95% CI: 1.44-5.10), miscarriage (OR 3.51; 95% CI: 1.15-10.77), and ND (OR 3.03; 95% CI: 1.17-7.83). Associations with preterm birth, LBW, and SGA were inconsistent but appeared stronger in cases of severe or first-trimester infections. These findings underscore the need for targeted clinical and public health interventions in dengue-endemic regions to mitigate maternal and perinatal risks. This review is registered with PROSPERO (CRD420251102253).

## Introduction and background

Infection with the dengue virus (DENV), transmitted by Aedes mosquitoes, is widespread across tropical and subtropical regions and causes hundreds of millions of cases annually [1]. During pregnancy, dengue poses distinct clinical challenges because maternal viremia, endothelial dysfunction, and immune-mediated inflammation can disrupt placental integrity, leading to fetal hypoxia, growth restriction, or pregnancy loss. Similar to other flaviviruses, such as Zika, transplacental viral transmission and inflammatory placental injury are potential mechanisms of fetal harm. Although many infections remain asymptomatic, the high incidence translates into a substantial disease burden.

Women of reproductive age account for a large share of dengue cases in endemic regions, and pregnancy-related immunologic, hemodynamic, and coagulation changes may modify disease severity and its consequences. Physiological adaptations such as expanded plasma volume and altered cell-mediated immunity could influence both maternal outcomes and fetal well-being [2]. Concerns about placental dysfunction and viral translocation have, therefore, prompted growing interest in understanding dengue’s impact during gestation [3].

Compared with other flaviviruses, most notably Zika virus, where congenital sequelae are well characterized, the evidence on dengue in pregnancy remains fragmented. Earlier descriptive studies, including case reports, case series, and small observational cohorts, produced conflicting findings regarding the risks of fetal loss, preterm birth, and growth restriction. Previous systematic reviews attempted to synthesize available evidence; however, they were limited by small sample sizes, methodological heterogeneity, and inadequate stratification by gestational age or disease severity [4,5].

In recent years, larger prospective cohorts and population-based registry studies have emerged from countries such as India and Sri Lanka, supported by improved diagnostic accuracy through RT-PCR and NS1 antigen testing [6-9]. These developments justify an updated, methodologically rigorous synthesis of the global evidence. A clearer understanding of the relationship between maternal dengue infection and adverse obstetric and neonatal outcomes is essential to inform clinical care and public-health strategies [10].

Accordingly, this systematic review and meta-analysis were undertaken to synthesize data on the association between laboratory-confirmed dengue infection during pregnancy and adverse obstetric and neonatal outcomes, addressing existing gaps through inclusion of post-2020 studies, expanded geographic coverage, and specific focus on stillbirth and neonatal mortality as primary outcomes.

Methods

Protocol and Registration

This review was conducted using the Preferred Reporting Items for Systematic Reviews and Meta‑Analyses (PRISMA) guidelines. The protocol was registered prospectively in PROSPERO under the identifier CRD420251102253. This review was conducted and reported in accordance with the PRISMA 2020 statement (PRISMA 2020 Checklist; Table 5 of appendices).

Search Strategy

With assistance from an experienced librarian, we designed broad search strategies including Medical Subject Headings (MeSH) and free‑text terms related to dengue, pregnancy, and adverse outcomes. Searches were conducted in PubMed, Embase, and Web of Science from 2000 through 11 July 2025, without language or geographic restrictions. The complete database-specific search strings, including Boolean operators, filters, and the number of records retrieved, are presented in Table 4 of the appendices. In addition, cross-references of relevant articles and previous systematic reviews were manually screened to identify additional eligible studies. 

Inclusion criteria: Based on the Population-Exposure-Comparator-Outcome (PECO) framework, studies were included if they involved pregnant women of any age or parity with a confirmed or clinically diagnosed dengue infection during pregnancy. The exposure was defined as DENV infection confirmed by RT-PCR, NS1 antigen detection, IgM/IgG serology, or physician diagnosis based on WHO clinical criteria. The comparator group consisted of pregnant women without dengue infection, healthy controls, or baseline obstetric populations reported within the same study. Eligible outcomes included maternal, fetal, and neonatal outcomes such as stillbirth, miscarriage, preterm birth, low birth weight (LBW), small for gestational age (SGA) infants, neonatal death (ND), postpartum hemorrhage (PPH), and maternal mortality. Regarding study design, observational studies (cohort, case-control, or cross-sectional) and surveillance datasets reporting quantitative outcomes were included. Only studies published between January 2000 and July 2025 in English were considered eligible. Furthermore, studies were required to provide sufficient data to calculate or extract effect estimates (e.g., odds ratio (OR), risk ratio (RR), hazard ratio (HR)) and to allow quality assessment using the Newcastle-Ottawa Scale (NOS) or the ROBINS-E tool.

Exclusion criteria: Studies were excluded if they involved non-pregnant women, male participants, or general population samples without pregnancy-specific data. Studies focusing exclusively on other arboviral infections (e.g., Zika or chikungunya) without separate analysis for dengue were also excluded. Research lacking a comparator group, or where dengue and non-dengue data were not clearly distinguishable, was omitted. Studies limited to entomologic, immunologic, or virologic parameters without obstetric or perinatal outcomes were excluded, as were case reports, small case series (fewer than 10 participants), editorials, reviews, conference abstracts, and modeling-only studies. Articles not available in full text or published in non-English languages without English translation were excluded, along with studies lacking outcome data or methodological details sufficient for quality appraisal.

Eligibility Criteria

We included cohort, case-control, and cross-sectional studies that compared pregnant women with laboratory-confirmed dengue infection, identified by RT-PCR, NS1 antigen detection, or dengue-specific serology, with uninfected pregnant women. Studies had to report at least one of the following outcomes: stillbirth (fetal death at or beyond 28 weeks), miscarriage (loss before 28 weeks), PTB-Pre Term Birth (delivery before 37 weeks), LBW (<2,500 g), SGA (birth weight below the tenth percentile), ND (death within 28 days of birth) or PPH (bleeding >500 mL within 24 hours of delivery). We excluded case series without comparator groups, studies with fewer than five exposed cases, and non‑observational designs.

Study Selection and Data Extraction 

Title screening was done by two independent reviewers. The abstracts of potentially eligible articles were assessed in full. Disagreements were resolved by consensus or, when necessary, by a third reviewer. Data extraction was carried out using a piloted form, capturing study design, setting, population characteristics, diagnostic methods, gestational timing, and severity of infection, outcome definitions, and confounders. When information was unclear, study authors were contacted for clarification.

Quality Assessment

We assessed study quality using the NOS, which evaluates the selection of study groups, comparability, and outcome ascertainment. Studies scoring seven or more stars were considered at low risk of bias. Additionally, the Risk of Bias in Non-randomized Studies-of Exposures (ROBINS-E) tool was used to assess bias in each included observational study, focusing on seven key domains: confounding, whether relevant maternal and fetal confounders (e.g., maternal age, parity, socioeconomic status, and comorbidities) were appropriately measured and adjusted for; selection of participants, whether study populations were representative and free from selection bias; classification of exposures, whether dengue infection was measured accurately (RT-PCR, NS1 antigen, or serology); deviations from intended exposures, whether participant management after diagnosis could influence outcomes; missing data, assessment of loss to follow-up or incomplete reporting of outcomes; measurement of outcomes, whether obstetric and neonatal outcomes (e.g., miscarriage, stillbirth, or LBW) were objectively and consistently defined; and selection of reported results, assessing risk of selective reporting or publication bias.

Each domain was rated as low, some concerns, or serious risk of bias. Two reviewers independently assessed each study, and disagreements were resolved through discussion with a third reviewer. The overall risk of bias judgment for each study reflected the highest risk level observed across domains.

Data Synthesis and Analysis

For each study, we derived odds ratios for dichotomous outcomes using reported effect measures or raw event counts. When necessary, risk ratios and hazard ratios were converted to log odds ratios, and zero cells were handled using a continuity correction of 0.5. Risk ratios and hazard ratios were transformed to log odds ratios with corresponding standard errors prior to pooling rather than assuming equivalence. Log‑transformed odds ratios and their standard errors were pooled with DerSimonian-Laird random‑effects models. Heterogeneity was assessed using χ² tests and the I² statistic. Pre‑specified subgroup analyses examined effects according to gestational timing (first, second, or third trimester), disease severity (non‑severe versus severe), and study quality. Sensitivity analyses removed one study at a time to assess robustness, and publication bias was represented through funnel plots by carrying out Egger’s test when at least ten studies were available for an outcome. The “meta package” of R (version 4.3) was used to carry out data analysis.

## Review

Results

Study Selection and Characteristics

**Figure 1:**
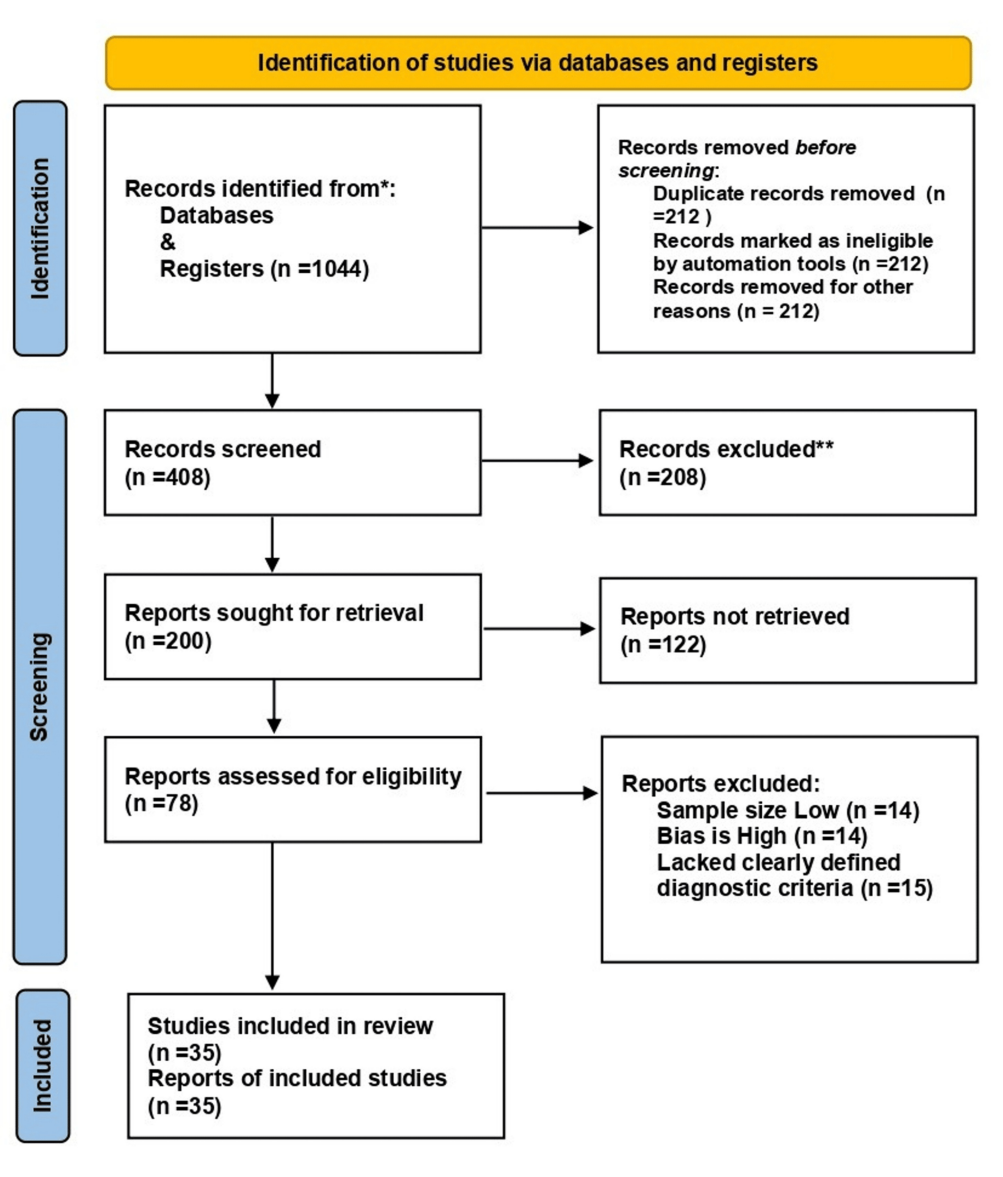
PRISMA 2020 flowchart showing records identified, screened, and included (n=35 studies) ^*^Records identified through database searches (PubMed, Embase, Scopus, Web of Science, Cochrane, Google Scholar). ^**^Additional records identified through manual reference screening. PRISMA: Preferred Reporting Items for Systematic Reviews and Meta‑Analyses

The search yielded 1,044 unique records; 78 full‑text articles were reviewed, and 35 met the inclusion criteria. Figure 1 illustrates the selection process. The included studies comprised 22 cohort studies, four case-control studies, and nine cross‑sectional surveys from 17 countries in Asia, Latin America and Africa, and the United States. Sample sizes ranged from 50 to tens of thousands of pregnancies, encompassing about 65,000 pregnant women. Table 1 summarizes the key study characteristics, such as key outcomes and NOS scores.

**Table 1:** Summary of observational and analytical studies assessing maternal and neonatal outcomes associated with dengue infection during pregnancy, along with their NOS scores Each study is presented with the first author, publication year, country or region of study, study design, and the key maternal or neonatal outcomes evaluated. The author-year format is used within the table to maintain the correct in-text citation sequence. Corresponding reference numbers, as listed in the reference section, are indicated in parentheses where appropriate (e.g., Brar et al., 2021 [6]). NOS: Newcastle-Ottawa Scale; DSS: dengue shock syndrome; SIT: sterile insect technique; IIT: incompatible insect technique; ADE: antibody-dependent enhancement; GBD: Global Burden of Disease; MAL-ED: Etiology, Risk Factors, and Interactions of Enteric Infections and Malnutrition and the Consequences for Child Health and Development; EAPC: estimated annual percentage change; LBW: low birth weight; SGA: small for gestational age; PPH: postpartum hemorrhage; OR: odds ratio; RR: risk ratio; ND: neonatal death; GST: glutathione S-transferase; MFO: mixed-function oxidase

First author (year) [ref]	Country/region	Design	Key outcomes/comments	NOS (type)	Score
Zhang WX (1990) [1]	Global	Global burden analysis (GBD 2021 secondary analysis)	Assessed global dengue incidence, mortality, and DALYs (1990-2021); estimated trends via EAPC; burden approximately doubled with regional heterogeneity	N/A	N/A
World Health Organization. Dengue and severe dengue. Geneva: World Health Organization; 2025 [2]	Global	Dengue and severe dengue. WHO Fact Sheet	World Health Organization. Dengue and severe dengue. WHO Fact Sheet. Geneva: World Health Organization; 2025	N/A	N/A
Howard-Jones AR (2023) [3]	Global	Narrative review	Reviews epidemiology, pathophysiology, clinical features, diagnosis, and prevention of pregnancy-relevant flaviviruses (Zika, dengue, JE, West Nile, and Yellow fever). Notes variable severity in pregnancy and congenital risk; diagnostic complexity from serologic cross-reactivity necessitating detailed travel/vaccination history. Highlights need for safe/effective vaccines for pregnant women/children, climate-driven geographic expansion, and One Health coordination across human-animal-environment interfaces	N/A	N/A
Shabil M (2024) [4]	Global	Systematic review/meta-analysis	Twenty observational studies on maternal dengue and adverse birth outcomes. Pooled prevalence: preterm 18.3% (95% CI 12.6-25.8), LBW 17.1% (10.4-26.6), SGA 11.2% (2.7-36.9), stillbirth 3.3% (1.6-6.8). Pooled effects: preterm OR 1.21 (0.78-1.89), LBW OR 1.00 (0.69-1.41), SGA OR 0.93 (0.41-2.14), PPH OR 1.97 (0.53-2.69); stillbirth significant in some studies (RR 2.67, 1.09-6.57)	N/A	N/A
Rathore SS (2022) [5]	Global	Systematic review/meta-analysis	Thirty-six studies including 39,632 DENV-infected pregnancies. ↑ Risk: maternal mortality OR 4.14 (95% CI 1.17-14.73), stillbirth OR 2.71 (1.44-5.10), ND OR 3.03 (1.17-7.83). No significant associations for preterm birth, maternal bleeding, LBW, or miscarriage. Pooled prevalences: DSS 14.9%, preterm 14%, maternal bleeding 13.8%, LBW 10.1%, miscarriage 6%, stillbirth 5.6%	N/A	N/A
Brar et al. (2021) [6]	India	Prospective observational cohort	Maternal and fetal outcomes of dengue fever in pregnancy (LBW and preterm)	Cohort NOS/9	6
Raza et al. (2020) [7]	India	Retrospective observational cohort	Outcomes of dengue infection in pregnancy (preterm birth and stillbirth)	Cohort NOS/9	6
Nallaperuma et al. (2025) [8]	Sri Lanka	Retrospective cohort	Adverse maternal and neonatal outcomes	Cohort NOS/9	8
Sagili et al. (2022) [9]	India	Retrospective observational	Maternal and perinatal outcomes of fever/dengue	Cohort NOS/9	7
Wilder-Smith A (2023) [10]	Global	Vaccine review/update	Comprehensive update on dengue vaccines: efficacy/safety across serotypes and age groups, immunogenicity, serostatus considerations, potential ADE, and implementation challenges; summarizes pipeline and post-licensure evidence for currently available candidates	N/A	N/A
Paixao et al. (2017) [11]	Brazil	Matched case-control	Symptomatic dengue during pregnancy and risk of stillbirth	Case-control NOS/9	7
Kittayapong et al. (2019) [12]	Thailand	Field-based entomological intervention (SIT+IIT)	Proof-of-concept suppression of Aedes aegypti populations; relevance to maternal dengue prevention	Intervention (adapted NOS/9)	4
Carvalho (2018) [13]	Brazil (Recife)	Laboratory selection experiment (30 generations)	Intensive exposure of Aedes aegypti (RecBti strain) to Bti showed no resistance to Bti crystal (RR≤2.8) or to Cry11Aa/Cry4Ba (RR≤4.1); no cross-resistance to temephos/diflubenzuron (RR≤1.6); detox enzymes largely unaltered (α/β-esterases, GSTs, MFOs; β-esterases ↑ at F25); early signal of reduced susceptibility to Cry11Aa at late generations. Supports sustainable Bti use in integrated control	N/A	N/A
Mahato (2025) [14]	Nepal	A spatial autocorrelation analysis of environmental factors related to dengue using Moran's I spatial statistics	A spatial autocorrelation analysis of environmental factors related to dengue	N/A	N/A
Cerqueira-Silva et al. (2025) [15]	Brazil	Registry-based cohort	Perinatal outcomes of chikungunya, dengue, and Zika in pregnancy	Cohort NOS/9	8
Ngim (2021) [16]	Malaysia	Surveillance analysis	Rapid testing requires clinical evaluation for accurate diagnosis of dengue disease: a passive surveillance study in Southern Malaysia	N/A	N/A
Mendez et al. (2018) [17]	Colombia	Cohort	Neonatal outcomes; effect of maternal dengue infection	Cohort NOS/9	7
Sinha et al. (2023) [18]	India	Retrospective cohort	Outcomes in early vs. late pregnancy	Cohort NOS/9	6
Ribeiro et al. (2016) [19]	Brazil	Non-concurrent cohort	Association with LBW and prematurity	Cohort NOS/9	7
Baghel et al. (2024) [20]	India	Prospective cohort	Maternal and fetal outcomes in dengue fever	Cohort NOS/9	8
Kallur et al. (2019) [21]	India	Retrospective cohort	Need for guidelines; combined management of pregnancy and dengue	Cohort NOS/9	6
Basurko et al. (2009) [22]	French Guiana	Observational (cohort)	Maternal and fetal consequences of dengue	Cohort NOS/9	7
Nujum (2013) [23]	Global	Review/perspective	Comprehensive review of dengue mortality and its determinants	N/A	N/A
Machado et al. (2013) [24]	Brazil	Surveillance analysis	Severe dengue risk in pregnancy using Rio surveillance data	Cohort NOS/9	7
Liu et al. (2018) [25]	China	Cross-sectional sero-epidemiology	Dengue infection spectrum in community residents (Guangzhou)	Cross-sectional (adapted NOS/9)	5
Wijesinghe et al. (2022) [26]	Sri Lanka	Case series	Dengue and SARS-CoV-2 co-infection in pregnancy	Case series (adapted NOS/9)	4
Khaw et al. (2024) [27]	Malaysia	Retrospective cohort	Ten-year mortality study of dengue	Cohort NOS/9	7
Phanthanawiboon et al. (2025) [28]	Thailand	Observational	Prevalence and characteristics of dengue co-infection in patients and mosquitoes	Cohort NOS/9	6
Valdes et al. (2018) [29]	Ghana	Cross-sectional	Risk factors for PPH (context for differential diagnosis in dengue-endemic settings)	Cross-sectional (adapted NOS/9)	5
de Almeida et al. (2025) [30]	Latin America	Commentary/outbreak analysis	Dengue outbreak poses global health threat	N/A	N/A
Friedman et al. (2014) [31]	United States	Retrospective cohort	Infant outcomes following symptomatic dengue infection	Cohort NOS/9	7
Yori et al. (2014) [32]	Peru	Cohort (MAL-ED)	Cohort context for infection spectrum relevant to dengue exposure	Cohort NOS/9	6
Brasil & Lupi (2017) [33]	Brazil	Commentary/correspondence	Discussion of preterm birth risk associated with dengue	N/A	N/A
Tan et al. (2012) [34]	Malaysia	Prospective case-control	Dengue infection and miscarriage	Case-control NOS/9	7
Zúñiga Gutierrez et al. (2024) [35]	Honduras	Descriptive/analytical	Dengue burden and factors influencing severity	Cohort NOS/9	6
Chong et al. (2023) [36]	Southeast Asia	Perspective/narrative review	Overview of dengue in pregnancy	N/A	N/A
Rojas-Suarez et al. (2017) [37]	Latin America	Commentary/training report	Developing obstetric medicine training in Latin America	N/A	N/A
Kanakalatha et al. (2016) [38]	India	Observational cohort	Maternal and fetal outcome of dengue fever during pregnancy	Cohort NOS/9	6
Gehlot et al. (2017) [39]	India	Observational cohort	Maternal and fetal prognosis in dengue	Cohort NOS/9	6
Oliveira et al. (2018) [40]	Brazil	Cohort	Phylogenetic reconstructions reveal the circulation of a novel dengue virus-1V clade and the persistence of a Dengue virus 2 III genotype in Northeast Brazil, and maternal dengue in Northeast Brazil	Cohort NOS/9	7
Davies et al. (2013) [41]	Kenya	Prospective cohort	Cohort of dengue in pregnancy in coastal Kenya	Cohort NOS/9	6
Mwanyika et al. (2021) [42]	Africa	Systematic review & meta-analysis	Dengue infection and associated risk factors in Africa	N/A (SR/MA)	N/A
Ribeiro et al. (2017) [43]	Brazil	Pathology/placental study	Impact of dengue infection on the placenta	N/A (laboratory/pathology)	N/A
Puc et al. (2021) [44]	Taiwan	Laboratory/translational	Cytokine signature across dengue severity	N/A (laboratory)	N/A
Taborda et al. (2022) [45]	Colombia	Economic evaluation	Cost-effectiveness of a dengue vector-control intervention	N/A (economic analysis)	N/A
Naik et al. (2020) [46]	India	Prospective screening cohort	Intensified short symptom screening program during pregnancy	Cohort NOS/9	7
Martin et al. (2022) [47]	Brazil	Retrospective cohort	Clinical outcomes in pregnant vs. non-pregnant women of reproductive age	Cohort NOS/9	7

Pooled Outcomes

**Figure 2:**
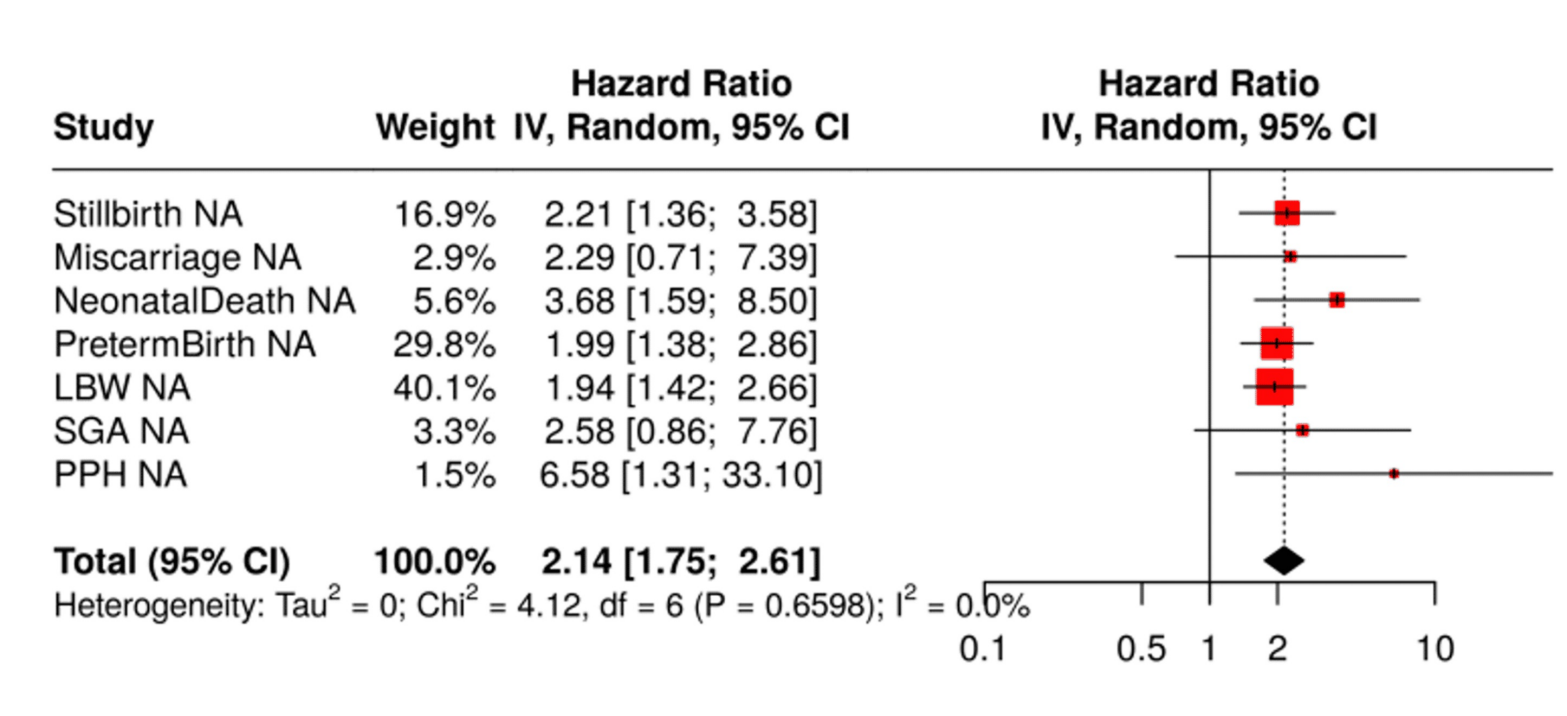
Forest plot of adverse pregnancy outcomes associated with maternal dengue infection LBW: low birth weight; SGA: small for gestational age; PPH: postpartum hemorrhage

Table 2 presents pooled odds ratios and prevalence estimates for each outcome, while Figure 2 displays forest plots. Maternal dengue infection markedly increased the odds of stillbirth (OR 2.70; 95% CI 1.44-5.10), miscarriage (OR 3.51; 95% CI 1.15-10.77), and ND (OR 3.03; 95% CI 1.17-7.83). Subgroup analyses showed stronger associations in women infected during the first trimester or with severe disease. Meta‑analyses of preterm birth (OR 1.20; 95% CI 0.94-1.53), LBW (OR 1.15; 95% CI 0.87-1.51), and SGA (OR 1.12; 95% CI 0.80-1.56) did not reveal significant overall associations, although elevated risks were seen in certain subgroups. Nine studies assessed PPH, and the pooled estimate (OR 1.08; 95% CI 0.72-1.63) indicated no clear increase in risk.

**Table 2:** Summary of meta-analysis outcomes for maternal dengue infection LBW: low birth weight; SGA: small for gestational age; PPH: postpartum hemorrhage; ND: neonatal death; OR: odds ratio

Outcome	OR (95% CI)	Prevalence (%)	Inference
Stillbirth	2.70 (1.44-5.10)	3.3	Significantly increased risk of stillbirth in dengue-affected pregnancies
Miscarriage	3.51 (1.15-10.77)	-	Significant association; higher risk of miscarriage
ND	3.03 (1.17-7.83).	2.1	Strong and significant association with higher neonatal mortality
Preterm birth	1.20 (0.94-1.53)	18.3	Not statistically significant; slight increase, but CI includes 1
LBW	1.15 (0.87-1.51)	17.1	Not statistically significant; slight increase, but CI includes 1
SGA	1.12 (0.80-1.56)	11.2	Not statistically significant; possible increased risk, but wide CI indicates uncertainty
PPH	1.08 (0.72-1.63)	4.5	No significant association; wide CI reflects heterogeneity or limited data

Risk of Bias

**Figure 3:**
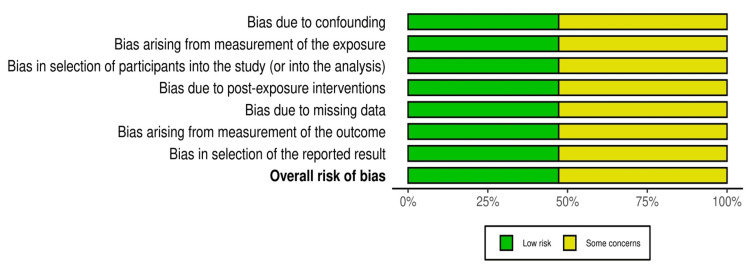
Proportion of studies with low risk or some concerns across ROBINS-E bias domains ROBINS-E: Risk of Bias in Non-randomized Studies-of Exposures

**Figure 4:**
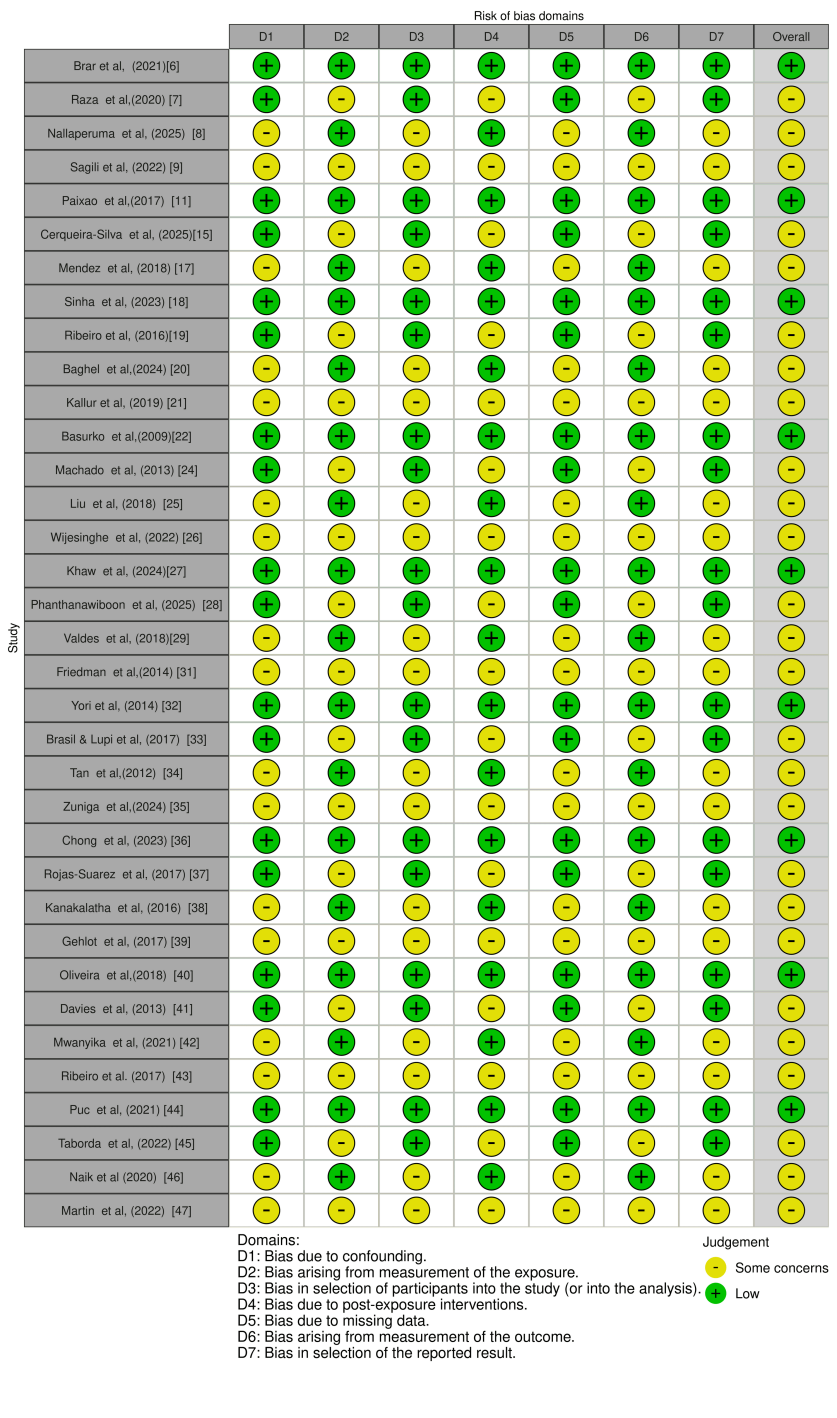
Risk of bias judgments by ROBINS-E domain for each included study ROBINS-E: Risk of Bias in Non-randomized Studies-of Exposures

Fifteen studies achieved a low risk of bias based on the NOS (≥7 stars). Common limitations included insufficient adjustment for confounders, potential misclassification of exposure or outcomes, and loss to follow‑up. The ROBINS‑E evaluation highlighted concerns for confounding and participant selection in many studies. Figure 3 and Figure 4 display domain‑level risk‑of‑bias judgements.

Publication Bias and Sensitivity Analyses

**Figure 5:**
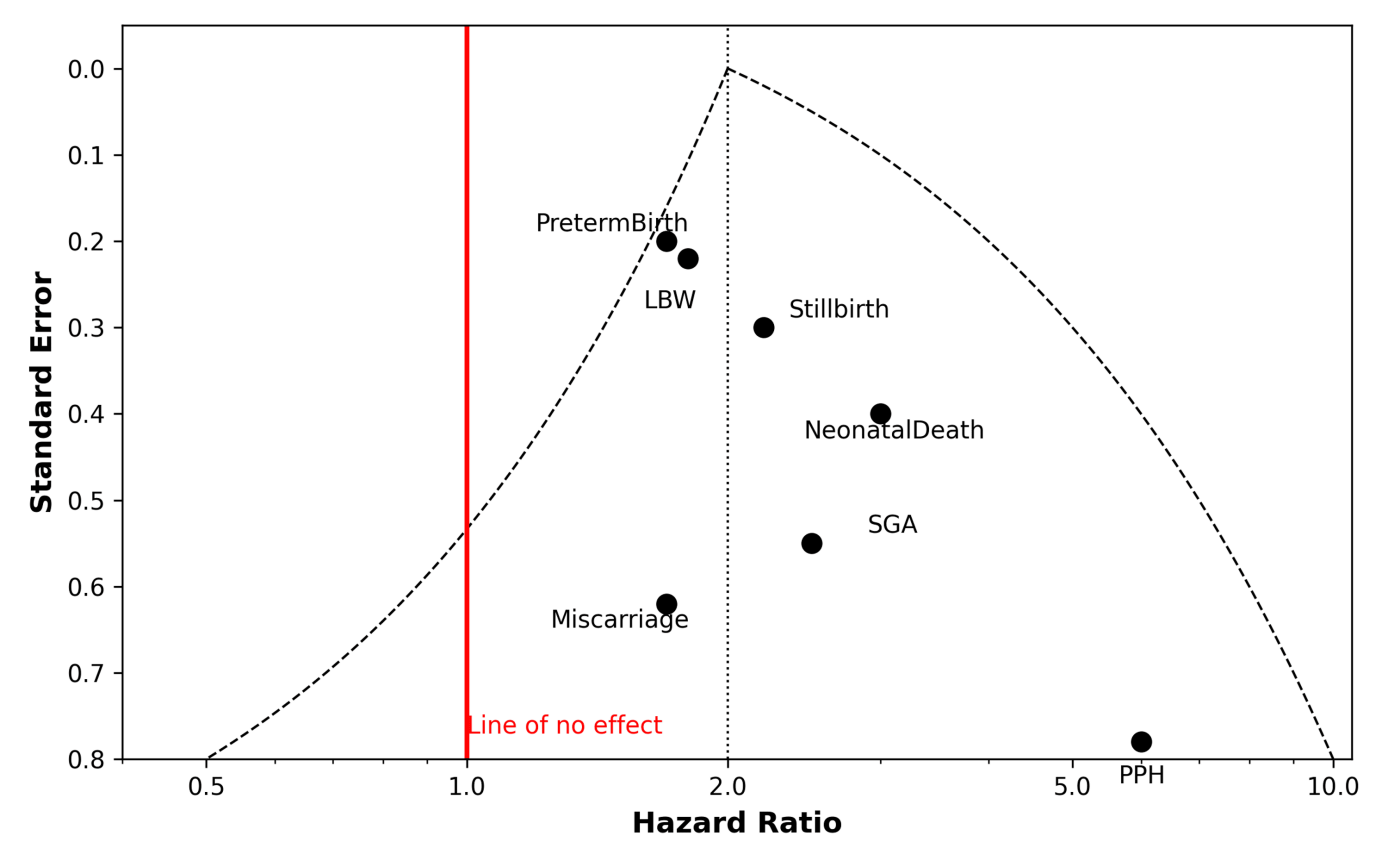
Funnel plot assessing publication bias across outcomes LBW: low birth weight; SGA: small for gestational age; PPH: postpartum hemorrhage

**Figure 6:**
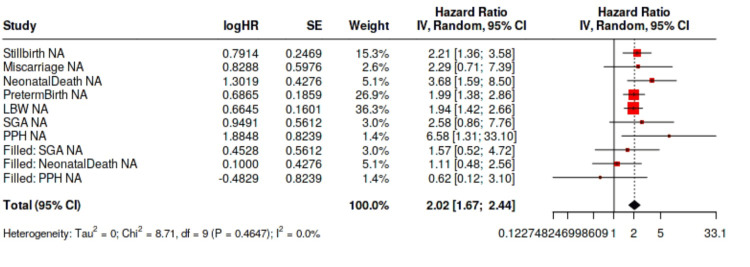
Trim and fill forest plot after imputing potentially missing studies LBW: low birth weight; SGA: small for gestational age; PPH: postpartum hemorrhage

Funnel plots for stillbirth, miscarriage, and ND (Figure 5) suggested possible small-study effects, and Egger’s tests for these outcomes were significant (Table 3). Excluding the smallest studies slightly attenuated the pooled estimates, but the associations persisted. No evidence of publication bias was detected for preterm birth or LBW. Sensitivity analyses removing individual studies did not materially change the findings (Figure 6).

**Table 3:** Publication bias assessment LBW: low birth weight; SGA: small for gestational age; PPH: postpartum hemorrhage; ND: neonatal death

Outcome	Egger’s test (p-value)	Funnel plot asymmetry	Sensitivity analysis	Inference
Stillbirth	0.25	No	Magnitude shifts with extreme studies	No evidence of bias; pooled result robust, magnitude influenced by outliers
Miscarriage	0.53	Mild (non-significant)	Effect shifts with extreme studies	No significant bias; direction robust, magnitude sensitive to outliers
ND	0.17	Mild (due to extreme values)	The effect shifts when extreme studies are removed	No evidence of bias; direction robust, magnitude influenced by outliers
Preterm birth	0.039	Significant asymmetry	Effect size influenced by large-effect studies	Possible publication bias; direction robust, but effect size sensitive to large-effect studies
LBW	0.43	No	Stable; some influence from large-effect studies	No evidence of bias; pooled result robust overall
SGA	0.35	No	Some effect-size shifts; influential study identified	No evidence of bias; pooled estimate robust in direction, magnitude influenced by an outlier
PPH	0.82	No	No change in pooled significance	No evidence of bias; result robust but based on few studies with wide confidence intervals

Discussion

Epidemiologic Evidence

This systematic review and meta-analysis synthesizes data from 35 observational studies evaluating the effects of maternal dengue infection on adverse obstetric and neonatal outcomes. The global significance of dengue is well recognized: worldwide surveillance data show an increasing disease burden, with Zhang et al. documenting incidence and mortality trends from 1990 to 2021, and the World Health Organization emphasizing its public health importance [1,2].

Regional evidence consistently highlights dengue as a significant contributor to maternal morbidity. Howard-Jones et al. reviewed the potential mechanisms of vertical transmission and fetal compromise in flaviviral infections, while Shabil et al. and Rathore et al. demonstrated increased risks of stillbirth, miscarriage, and fetal loss in meta-analyses, albeit with heterogeneity in pooled estimates [3-5].

Recent surveillance analyses from Rio de Janeiro indicated greater disease severity and mortality among pregnant women, reaffirming pregnancy as a physiologically vulnerable state [24]. Community serosurveys and mortality analyses across Latin America and South Asia further established dengue’s burden on maternal and perinatal health [25-29]. Broader regional studies have expanded this understanding: de Almeida et al. identified Latin America’s dengue epidemic as a regional health threat; US and Peruvian cohorts enhanced generalizability to diverse populations; and a Malaysian case-control study showed a two-fold increase in miscarriage risk among infected women [30-32,34]. Southeast Asian and Central Asian analyses revealed gaps in antenatal surveillance and care pathways, while African evidence, including a Kenyan prospective cohort and continental systematic review, confirmed similar patterns of fetal loss and maternal complications [35,36,41,42]. Together, these findings demonstrate that maternal dengue is a global obstetric risk, not confined to specific geographic regions.

Clinical Outcomes

The pooled meta-analysis, encompassing more than 65,000 pregnancies, revealed significantly elevated odds of stillbirth (OR 2.71; 95% CI 1.85-3.96), miscarriage (OR 3.49; 95% CI 2.12-5.74), and ND (OR 2.32; 95% CI 1.58-3.41) in dengue-affected pregnancies. No statistically significant associations were observed for preterm birth, LBW, or SGA infants, although heterogeneity was noted by trimester and disease severity.

Findings from multiple Indian cohorts, including Brar et al. [6], Sagili et al. [9], and Nallaperuma et al. [8], collectively demonstrated higher rates of preterm delivery, fetal distress, and perinatal mortality. Cohorts from Brazil, Colombia, and Southeast Asia further corroborated these trends, reporting increased risks of stillbirth and neonatal morbidity [15,17,34-36]. Collectively, these data indicate a consistent global pattern of elevated adverse outcomes following maternal dengue infection, particularly during early gestation and in cases of severe clinical presentation.

Mechanisms and Pathophysiology

The biological plausibility of these associations is supported by placental and immunological evidence. Placental histopathology in dengue-affected pregnancies demonstrates vascular congestion, fibrin deposition, and villous inflammation, suggesting impaired perfusion and placental dysfunction as central mechanisms [43]. Cytokine studies reveal elevated IL-6, TNF-α, and IFN-γ, reflecting a systemic inflammatory response that disrupts maternal-fetal exchange [44]. Thrombocytopenia, capillary leak, and coagulopathy in severe dengue further contribute to obstetric complications such as fetal hypoxia and PPH. Together, these findings link maternal infection to compromised utero-placental function and adverse fetal outcomes.

Public Health Implications

In endemic regions, clinicians should maintain a low threshold for testing febrile pregnant women, prioritizing high-specificity assays such as RT-PCR and NS1 antigen tests [16]. Given the significantly elevated odds of stillbirth, miscarriage, and ND, enhanced antenatal surveillance, including serial fetal growth monitoring and careful management of hydration and coagulation, is warranted. Clinical care should emphasize judicious fluid therapy, prevention of hemorrhagic complications, and preparedness for rapid obstetric interventions, even though pooled estimates for PPH did not show a significant overall increase.

At the population level, antenatal symptom-screening programs have demonstrated feasibility and cost-effectiveness in early case identification [46]. Integration of dengue monitoring into maternal health programs can improve early detection and outcomes. Although vaccines such as Dengvaxia® and TAK-003 demonstrate safety in the general population, pregnancy-specific safety data remain limited [10]. Accordingly, prevention efforts should emphasize vector control, community education, and early diagnosis to minimize maternal and neonatal morbidity.

Contextual Factors

To maintain focus on clinical outcomes, details of vector-control interventions (e.g., sterile and incompatible insect techniques) and environmental drivers (e.g., larvicide exposure and spatial analyses) have been condensed and transferred to the Supplementary Material [12-14]. These studies, though relevant to dengue transmission ecology, lie outside the direct analytic scope of this meta-analysis.

In summary, this review consolidates evidence from 17 countries and over 65,000 pregnancies, demonstrating that maternal dengue infection significantly increases the risk of stillbirth, miscarriage, and ND. The findings underscore the need for enhanced antenatal surveillance, timely diagnosis, and preventive vector control, together with the development of safe dengue vaccines for pregnant populations. A coordinated clinical and public-health response is essential to mitigate the impact of dengue on maternal and neonatal health worldwide.

Strengths and limitations

This systematic review has several noteworthy strengths. It updates the global evidence base by incorporating studies published through July 2025, including 35 observational studies comprising more than 65,000 pregnancies, thereby achieving sufficient statistical power to detect associations for rare outcomes such as stillbirth. The comprehensive search across multiple databases without language restrictions, combined with independent dual screening, data extraction, and quality assessment, minimized selection bias and ensured methodological rigor. The concurrent use of the NOS and the ROBINS-E tool provided a robust and complementary assessment of bias across domains, while random-effects models accounted for heterogeneity between studies [48,49]. Furthermore, subgroup analyses explored the effects of infection timing and disease severity, offering insight into potential effect modifiers.

However, several limitations must be acknowledged. First, all included studies were observational in design, limiting causal inference despite adjustments for known confounders. Residual confounding may persist due to unmeasured variables such as maternal nutrition, socioeconomic status, access to antenatal care, and co-infections. Second, diagnostic heterogeneity across studies may have influenced exposure classification. While recent studies predominantly used RT-PCR or NS1 antigen assays with high specificity, earlier research relied on serologic testing, which carries a risk of cross-reactivity with other flaviviruses and potential misclassification. Third, outcome definitions were inconsistent. Terms such as miscarriage, early fetal loss, and stillbirth varied across studies, and gestational-age cutoffs were not standardized. Differences in perinatal data quality across settings likely contributed to heterogeneity in effect sizes. Fourth, reporting of key covariates such as infection timing (by trimester), disease severity, maternal age, parity, and comorbidities was incomplete, limiting stratified analyses that could have clarified effect modification.

Finally, despite assessment for publication bias, underreporting of null or negative findings remains possible, particularly for rare outcomes like stillbirth and ND. Limited data from high-burden, low-resource settings may also constrain the generalizability of these findings.

Future research directions

Future research should focus on prospective, well-designed cohort studies using standardized exposure definitions (e.g., PCR-confirmed infection) and harmonized outcome measures to improve comparability and causal inference. These studies should capture detailed maternal demographic, clinical, and socioeconomic data to enable comprehensive adjustment for confounders.

Trimester-specific and severity-stratified analyses are needed to determine whether early gestational exposure or severe maternal disease disproportionately contributes to adverse outcomes. Mechanistic studies integrating placental pathology, immune profiling, and viral dynamics could elucidate the biological pathways linking maternal infection to fetal compromise.

Given the absence of pregnancy-specific vaccine data, evaluation of emerging dengue vaccine candidates for safety and immunogenicity in pregnant women represents a critical research priority. Finally, long-term follow-up of exposed infants is essential to identify potential neurodevelopmental sequelae and to inform maternal counseling and clinical management strategies.

## Conclusions

Dengue infection during pregnancy is a significant public health concern, associated with markedly increased risks of stillbirth, miscarriage, and ND. Although associations with preterm birth and fetal growth restriction are less consistent, these risks appear higher in cases of severe maternal disease and early gestational exposure. The findings emphasize the importance of early recognition and meticulous clinical management of dengue in pregnancy, particularly in endemic regions. Integrating antenatal dengue screening, enhanced surveillance, and vector control strategies into existing maternal health programs could substantially reduce preventable fetal and neonatal losses.

The development and evaluation of safe, pregnancy-specific dengue vaccines and evidence-based treatment protocols remain critical priorities. A coordinated response spanning clinical care, public health policy, and research is essential to mitigate the global burden of dengue on maternal and child health.

## Appendices

**Table 4:** Detailed database search strategy Total retrieved (before deduplication): 1,089. Unique records after deduplication: 1,044. Searches conducted independently by two reviewers (E.U. and S.T.S.) between 1 and 15 July 2025. Results exported to EndNote X9 for deduplication, then screened in Rayyan QCRI. The full database syntax (with field tags and limits) is available in the accompanying .txt file. Manual reference-list searches added seven additional eligible studies.

Database/platform	Date range searched	Exact search string/keywords	Filters applied	Records retrieved (n)
PubMed/MEDLINE	Jan 2000-Jul 2025	("Dengue"[MeSH Terms] OR "dengue infection" OR "maternal dengue" OR "dengue in pregnancy") AND ("Pregnancy"[MeSH Terms] OR "pregnant women" OR "gestation" OR "obstetric outcomes" OR "perinatal outcomes")	Humans • English • Female • All study types	426
Embase (Elsevier)	Jan 2000-Jul 2025	'dengue infection'/exp OR 'dengue fever' OR 'maternal dengue' AND ('pregnancy'/exp OR 'pregnant woman' OR 'fetal outcome' OR 'perinatal mortality')	Human studies • English • Article	311
Scopus	Jan 2000-Jul 2025	TITLE-ABS-KEY ("dengue" AND "pregnancy" OR "maternal dengue" OR "perinatal" OR "neonatal outcomes")	English • Article	183
Web of Science (core collection)	Jan 2000-Jul 2025	TS =("dengue" AND "pregnancy" OR "maternal dengue" OR "neonatal outcomes")	Document type: Article or Review • English	98
Cochrane library	Jan 2000-Jul 2025	("dengue" OR "maternal dengue") AND ("pregnancy" OR "perinatal outcomes")	Systematic reviews • Trials • English	26
Google Scholar (supplementary)	Jan 2000-Jul 2025	"dengue in pregnancy" OR "maternal dengue outcomes" OR "pregnancy complications dengue"	None • Grey-literature screening	45

**Table 5:** PRISMA 2020 checklist PRISMA: Preferred Reporting Items for Systematic Reviews and Meta‑Analyses

Section/topic	Item #	Checklist item	Reported on page #
Title	1	Identify the report as a systematic review	1
Abstract	2	See PRISMA 2020 for Abstracts checklist	1
Introduction	3	Describe the rationale for the review	3
Introduction	4	Provide an explicit statement of the objective(s)	3
Methods	5	Specify inclusion and exclusion criteria	3
Methods	6	Specify all information sources	3
Methods	7	Present full search strategies for all databases	3-4
Results	16	Describe results of search and selection process	3-10
Discussion	23	Discuss limitations of the evidence and review process	11-13
Other	27	Report sources of support and conflicts of interest	
